# Association of Circular RNAs (circRNAs) in Hepatitis B Virus (HBV) Induced Hepatocellular Carcinoma (HCC): Emerging Diagnostic Biomarkers and Novel Therapeutic Targets

**DOI:** 10.34172/apb.025.46424

**Published:** 2025-12-22

**Authors:** Nalira Yaugoob, Kannan Subbaram, Razana Faiz, Sheeza Ali, Queen Alice Arul

**Affiliations:** ^1^School of Medicine, The Maldives National University, Male’, Maldives; ^2^All India Institute of Medical Sciences, Kalyani, India

## To Editor,

 Chronic hepatitis B affects about 5% of the global population, with the highest prevalence in Asia, and accounts for nearly 50% of all hepatocellular carcinoma (HCC) cases, including almost all childhood cases.^1-3^ Hepatitis B virus (HBV), a DNA virus of the*Hepadnaviridae* family, increases an individual’s lifetime risk of liver cancer by 15-20 fold.^2^ It produces oncogenic proteins such as hepatitis B virus x (Hbx) that drive hepatocarcinogenesis even in the absence of liver cirrhosis.^2^ With liver cancer incidence projected to rise by 55% by 2040, particularly in regions with high HBV burden, circular RNAs (circRNAs) have emerged as key regulators in HBV-related HCC, offering promise as novel biomarkers and therapeutic targets.^3^

**Figure 1:**
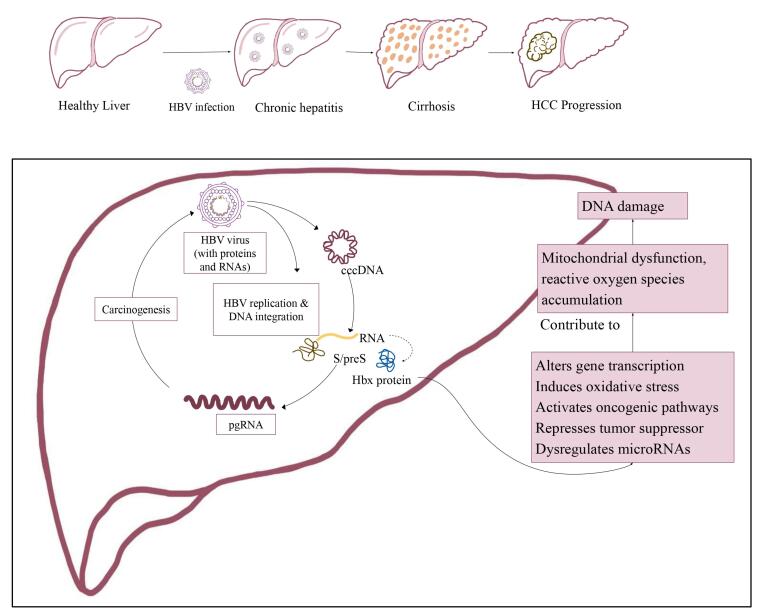
Pathways by which hepatitis B virus infection leads to liver cancer. The figure illustrates the progression of HBV infection from a healthy liver through chronic hepatitis and cirrhosis to HCC. The HBV drives carcinogenesis, as its cccDNA and HBV DNA integration lead to the production of the oncogenic HBx protein. These factors cause DNA damage and oxidative stress (from mitochondrial dysfunction). This alters gene transcription, activates oncogenic pathways, represses tumor suppressors, and dysregulates microRNAs, culminating in HCC

 At the molecular level, HBV drives hepatocarcinogenesis through several interconnected mechanisms that persistently destabilize the host cell and compromise genetic integrity.^2,4^ This process is centered on the persistence of the viral covalently closed circular DNA (cccDNA) in hepatocytes, which acts as a stable transcriptional reservoir, and the integration of HBV DNA into the host genome, inducing chromosomal instability and insertional mutagenesis.^3^ Hbx is a pleiotropic factor that dramatically alters host gene transcription: it potently activates major oncogenic pathways (such as Wnt/β-catenin, MAPK, NF-kB, and PI3K/Akt) while simultaneously repressing tumor suppressors and dysregulating microRNAs (miRNAs).^2^ The combined effect of these alterations, coupled with chronic oxidative stress, endoplasmic reticulum stress, and specific HBV genome mutations (particularly in PreS/S, P, PreC, and X genes), leads to multifactorial dysfunction, accumulation of reactive oxygen species (ROS), and resultant DNA damage (Figure 1).^4^

 Furthermore, the carcinogenic process is amplified by factors like viral load, HBeAg, and HBsAg, which foster a complex tumor microenvironment characterized by immune suppression.^4^ In this setting of chronic molecular assault, circRNAs - stable, covalently closed transcripts generated primarily by alternative splicing - become dysregulated.^2,3^ They can function as miRNA sponges, protein scaffolds, and transcriptional regulators.^2^ Notably, the virus itself encodes an oncogenic factor, HBV-circ 1, which promotes cell cycle progression and is independently linked to lower survival rates in positive patients.^5^

 Clinically, circRNAs offer significant promise as HBV-related cancer biomarkers due to their exceptional stability in biofluids, making them ideal for non-invasive diagnostics.^2^ Several studies have demonstrated that circulating circRNAs can complement or even surpass the diagnostic performance of α-fetoprotein (AFP), particularly in early stage or AFP negative disease. NotablyWu et al. (2020) identified three plasma circRNAs (circ_0009582, circ_0037120, circ_0140117) that, when combined with AFP, achieved an area under the curve (AUC) of 0.988 in the training set, significantly outperforming AFP alone (AUC 0.740) for HBV-HCC detection.^6^ Therapeutically, circRNAs are being explored as both targets and tools for novel interventions.^2^ Strategies include RNA interference (RNAi) to silence oncogenic circRNAs and the design of synthetic circRNAs for regulatory or vaccine applications, which utilize their stability and capacity to encode antigens efficiently via internal ribosomal entry sites (IRES) or m6A modification, allowing them to act as self-adjuvants that boost antitumor immunity.^2^ This circular structure, which resists exonuclease degradation, also supports their stable and effective incorporation into advanced drug delivery systems currently under investigation, such as lipid nanoparticles (LNPs), polymeric carriers, exosomes, and viral vectors.^2^ Furthermore, certain herbal extracts such as *Scutellaria barbata*and *Oldenlandia diffusa*have been reported to modulate circRNA networks, suggesting potential adjunctive strategies that could be developed alongside conventional treatments.^2^

## Conclusion

 In summary, circRNAs represent a novel frontier in HBV-HCC research, offering opportunities for early diagnosis, prognostic evaluation, and therapeutic intervention. Their stability, tissue specificity, and regulatory versatility, together with an improved understanding of HBV biology can contribute to its treatment. The Hbx mediated oncogenesis, immune microenvironment modulation, and emerging therapeutic approaches, could pave the way for personalized medicine strategies that improve outcome in this high-mortality malignancy. Future work should prioritize functional studies, exosomal circRNAs, circRNA-miRNA-mRNA networks, immune checkpoint regulation and delivery systems to fully harness their clinical potential.

## Competing Interests

 The authors declare that there are no conflicts of interest.

## Ethical Approval

 Not applicable.
